# Intergenerational Transmission of Psychiatric Conditions and Psychiatric, Behavioral, and Psychosocial Outcomes in Offspring

**DOI:** 10.1001/jamanetworkopen.2023.48439

**Published:** 2023-12-20

**Authors:** Mengping Zhou, Henrik Larsson, Brian M. D’Onofrio, Mikael Landén, Paul Lichtenstein, Erik Pettersson

**Affiliations:** 1 Department of Medical Epidemiology and Biostatistics, Karolinska Institutet, Stockholm, Sweden; 2 School of Medical Sciences, Örebro University, Örebro, Sweden; 3 Department of Psychological and Brain Sciences, Indiana University, Bloomington; 4 Institute of Neuroscience and Physiology, The Sahlgrenska Academy at Gothenburg University, Gothenburg, Sweden

## Abstract

**Importance:**

Psychiatric conditions in parents are associated with many psychiatric and nonpsychiatric outcomes in offspring. However, it remains unknown whether this intergenerational transmission is attributable to broader psychopathology comorbidity or to specific conditions.

**Objective:**

To estimate associations between general and specific psychopathology factors in parents and a wide range of register-based outcomes in their offspring.

**Design, Setting, and Participants:**

This Swedish national register-based cohort study included 2 947 703 individuals born between 1970 and 2000 and followed up with participants through December 31, 2013. Statistical analysis was performed from October 2022 to October 2023.

**Exposures:**

Hierarchical factor model consisting of 1 general and 3 specific psychopathology factors fit to 9 parental psychiatric diagnoses and violent criminal court convictions.

**Main Outcomes and Measures:**

A total of 31 outcomes were measured in offspring and sorted into 6 broad clusters: psychotic-like outcomes, neurodevelopmental outcomes, internalizing outcomes, externalizing outcomes, behavior and accidents, and psychosocial outcomes.

**Results:**

Of 2 947 703 individuals, 1 518 252 (51.5%) were male, and the mean (SD) age at the end of follow-up was 28.7 (8.9) years. The general psychopathology factor in parents was significantly associated with all 31 offspring outcomes (range: odds ratio [OR] for accidents, 1.08 [95% CI, 1.07-1.08] to OR for social welfare recipiency, 1.40 [95% CI, 1.39-1.40]), which means that children whose parents scored 1 SD above the mean on the general psychopathology factor had an 8% to 40% higher odds of different studied outcomes. The specific psychotic factor in parents was primarily associated with all 5 psychotic-like outcomes (range: OR for prescription of antiepileptics, 1.05 [95% CI, 1.04-1.06] to OR for schizophrenia, 1.25 [95% CI, 1.23-1.28]) and the specific internalizing factor in parents was primarily associated with all 6 internalizing outcomes (range: OR for prescription of anxiolytics, 1.10 [95% CI, 1.09-1.10] to OR for depression, 1.13 [95% CI, 1.12-1.13]) and all 6 neurodevelopmental outcomes (range: OR for intellectual disability, 1.02 [95% CI, 1.01-1.03] to OR for autism spectrum disorder, 1.10 [95% CI, 1.09-1.11]) in offspring. The specific externalizing factor in parents was associated with all 6 externalizing outcomes (range: OR for violent crimes, 1.21 [95% CI, 1.19-1.23] to OR for oppositional defiant disorder, 1.32 [95% CI, 1.32-1.33]) and all 6 internalizing outcomes (range: OR for obsessive-compulsive disorder, 1.01 [95% CI, 1.00-1.02] to OR for posttraumatic stress disorder, 1.13 [95% CI, 1.12-1.13]) in offspring.

**Conclusions and Relevance:**

This cohort study of the Swedish population suggests that the intergenerational transmission of psychiatric conditions across different types of spectra may largely be attributable to a parental general psychopathology factor, whereas specific factors appeared to be primarily responsible for within-spectrum associations between parents and their offspring. Professionals who work with children (eg, child psychologists, psychiatrists, teachers, and social workers) might benefit from taking the total number of parental psychiatric conditions into account, regardless of type, when forecasting child mental health and social functions.

## Key Points

**Question:**

Is the intergenerational transmission of psychiatric conditions attributable to broader psychopathology comorbidity or to specific conditions?

**Findings:**

In this cohort study including 2 947 703 participants, children whose parents scored 1 SD above the mean on the general psychopathology factor had a statistically significant 8% to 40% higher odds of 31 different outcomes. The specific psychopathology factors were primarily associated with within-spectrum and related offspring outcomes.

**Meaning:**

Because the intergenerational transmission of psychiatric conditions appeared largely attributable to a parental general psychopathology factor, mental health care professionals might benefit from taking the total number of parental psychiatric conditions into account when estimating patient prognosis.

## Teaser

This population-based cohort study estimates associations between general and specific psychopathology factors in parents and a wide range of register-based outcomes in their offspring.

## Introduction

Children of parents with psychiatric disorders have elevated probabilities for many psychiatric disorders^1,2,3^ and for behavioral and psychosocial problems.^4,5,6,7,8,9^ However, when adjusting for parental psychiatric comorbidity using multiple regression models, the unique parent-offspring associations tend to attenuate substantially.^2,7,8,10^ This finding suggests that adverse outcomes in offspring might be associated with the shared variance among parental psychiatric conditions, rather than with specific conditions.

Multivariate analyses suggest that 2 overarching spectra capturing internalizing (eg, depression) and externalizing problems (eg, harmful substance use) can account for the shared variance among psychiatric conditions.^11^ Studies indicate that some within-spectra intergenerational transmission can be attributed to these broader spectra (eg, parental internalizing problems associated with offspring internalizing problems).^12,13,14^ In addition, mirroring the fact that the internalizing and externalizing dimensions are substantially positively correlated,^15^ intergenerational transmission also occurs across these 2 spectra.^10^

Researchers have suggested that the shared variance among psychiatric dimensions may be better explained by a hierarchical factor model that consists not only of the internalizing and externalizing factors but also of a general psychopathology factor (the “p factor”).^16,17^ In contrast to multiple regression models, hierarchical factor models allow for simultaneously estimating the association of both shared variance (estimated via the general factor) and unique variance (estimated via specific factors that are unrelated to the general factor) of psychiatric conditions with a given outcome.^18^ This approach also enables researchers to explore whether a general psychopathology factor might account for the intergenerational transmission of psychiatric conditions across different spectra.

To our knowledge, only 2 studies have explored this possibility. First, in a Swedish children-of-twins study (N = 854), a general psychopathology factor based on self-reported internalizing and externalizing symptoms was positively associated between parents and their offspring.^19^ Second, in a Wisconsin twin study (N = 502), a parental internalizing factor was associated with an offspring general psychopathology factor based on interviewer-assessed internalizing and externalizing problems.^20^ Nevertheless, it remains unknown whether these results might be generalizable to other forms of mental health problems (eg, psychotic disorders) in parents and associated behaviors (eg, suicide and criminal convictions) in their offspring. In addition, neither of these studies quantified to what extent the transmission might be attributed to a general psychopathology factor. The aim of this study was to estimate associations between general and specific psychopathology factors in parents, and a wide range of psychiatric, behavioral, and psychosocial outcomes in their offspring.

## Methods

Ethical approval was granted by the regional ethical review board in Stockholm. No patient consent was obtained because this was a register-based epidemiologic study. This study complies with the Strengthening the Reporting of Observational Studies in Epidemiology (STROBE) reporting guideline.

### Participants

We identified all individuals born in Sweden between 1970 and 2000 from the Total Population Register and linked them to several national registries (eTable 1 in Supplement 1). All registers included follow-up until December 31, 2013, except the Prescribed Drug Register, which included follow-up until December 31, 2015. We excluded individuals for whom we could not identify both biological parents (5% of the total sample) and individuals who had congenital malformations, emigrated, or died (except for death from suicide) before December 31, 2013 (2 947 703 individuals left).

### Measures

#### Parental Psychiatric Conditions

The exposures were 9 psychiatric diagnoses in parents assigned by specialists, including schizophrenia, schizoaffective disorder, bipolar disorder, alcohol-related disorders, drug-related disorders, depression, anxiety, obsessive-compulsive disorder (OCD), and posttraumatic stress disorder (PTSD). All diagnoses were identified according to the *International Classification of Diseases, Eighth Revision* (*ICD-8*) (1969-1986), *International Classification of Diseases, Ninth Revision* (*ICD-9*) (1987-1996), and *International Statistical Classification of Diseases and Related Health Problems, Tenth Revision* (*ICD-10*) (1997-2009).^21^ We also included court convictions for violent crimes as a proxy for antisocial behavior. Parents were treated as having the condition (diagnoses or criminality) if either one was recorded in the registries.

#### Offspring Outcomes

We included 31 register-based outcomes in the offspring. To facilitate presentation, we sorted them into 6 theoretically oriented clusters roughly reflecting their shared medical and/or symptomatic origins. Psychotic-like outcomes included schizophrenia, bipolar disorder, and prescription of antipsychotics, lithium, and antiepileptics. Neurodevelopmental outcomes included attention-deficit/hyperactivity disorder, tic disorder, autism spectrum disorder, intellectual disability, learning disorders, and prescription of stimulants. Internalizing outcomes included anxiety, depression, PTSD, OCD, and prescription of anxiolytics and antidepressants. Externalizing outcomes included alcohol-related disorders, drug-related disorders, oppositional defiant disorder, court convictions for violent crimes, and prescription of antialcohol and antiopioid medications. The behavior and accidents cluster included suicide behavior, accidents, and violent victimization. The psychosocial outcomes included low school grade (defined as being ranked in the lowest quintile on the standardized junior high school grade measure), high school ineligibility, low cognitive ability (stanine [9-point scale], 1 [ie, <4%]), social welfare recipiency, and unemployment. Cognitive ability was measured by the Swedish Enlistment Battery (SEB) during the (male-only) mandatory military conscription evaluation. The SEB consisted of subtests measuring logical, spatial, verbal, and technical abilities, which were summarized into a total score and transformed to a 9-point scale with a mean (SD) of 5 (2). The mean (SD) time to complete the test was 62 (15) minutes.^22^ The SEB total score has high reliability and construct validity.^22,23^ eTable 2 in Supplement 1 presents the *ICD* code, a description of convictions classified as violent crimes, and the cutoff age for each exposure and outcome. All outcomes were treated as binary variables (ie, ever registered vs not).

#### Covariates

We included offspring birth year as a covariate to adjust for potential cohort and period effects. The highest parental educational level was included to adjust for socioeconomic status.

### Statistical Analysis

Statistical analysis was performed from October 22 to October 23. First, we conducted exploratory factor analysis (EFA), which allowed all 10 parental conditions to load on all factors (ie, cross-loadings were not constrained to zero). We decided on the number of factors to extract based on the scree plot, which contrasts the eigenvalues against the eigenvectors. We then rotated the extracted factors toward one general factor and several uncorrelated specific factors using a direct Schmid-Leiman (DSL) rotation,^24^ a so-called second-order hierarchical factor model (eAppendix in Supplement 1). To examine whether the hierarchical factor model varied by sex, we constructed the model separately for fathers and mothers. Because second-order models lack 1 degree of freedom when estimating associations with outcomes,^25^ we applied the DSL rotation within an exploratory structural equation modeling (ESEM) to simultaneously regress each of the offspring outcomes onto the latent factors^26^ (associated R and Mplus code shown in the eAppendix in Supplement 1). To account for the dependence of individuals, we estimated robust SEs by clustering on maternal identifier. We used the mean- and variance-adjusted weighted least-square estimator. To address the problem of multiple testing (n = 31 × 4), we corrected all *P* values using the Benjamini-Hochberg false discovery rate. *P* < .05 was considered statistically significant, and all tests were 2-tailed. To examine whether the results varied by offspring sex, we further analyzed the association separately by males and females in the offspring.

We also used 2 additional ways to compute the relative contribution of general and specific psychopathology factors to the associations. First, we decomposed the bivariate parental-offspring associations into that which might be attributed to the general vs specific psychopathology factors. Second, we decomposed the variance explained (ie, *R*^2^) in the outcomes into the relative proportions that might be attributable to the general vs specific psychopathology factors. eFigure 1 in Supplement 1 presents a graphical description for the calculation within the ESEM.

In sensitivity analyses, we first tested the robustness of the results by fitting different types of hierarchical models. To ensure that the associations between specific factors and offspring outcomes were not attributable to cross-loadings, we estimated a second-order confirmatory factor analysis model, in which we constrained the EFA-specific factors loading less than 0.40 at zero. To circumvent the issue with lacking 1 degree of freedom when estimating associations with outcomes, we constrained 1 specific factor to have zero residual, and therefore it would have no association with the outcomes (a detailed description and Mplus code shown in the eAppendix in Supplement 1). Furthermore, to ensure the results were not attributable to modeling a second-order hierarchical model, we also fit a bifactor EFA model, which identified 1 general factor that directly accounts for the shared variance among all 10 parental conditions and several specific factors that capture the unique variance unexplained by the general factor simultaneously.^27^

Second, to examine whether the results were robust to changing diagnostic criteria, we fit the same hierarchical models as above (second-order EFA, second-order confirmatory factor analysis) but limited the observed indicators to only 6 parental diagnoses for which the *ICD* criteria remained relatively constant over time (schizophrenia, bipolar disorder, depression, anxiety, alcohol-related disorders, and drug-related disorders).

Third, to avoid reverse causation, we fit the same second-order EFA model but excluded children whose parents have the condition after the child’s birth to ensure that the exposures preceded the outcomes. All analyses were conducted with the Mplus software (Muthén & Muthén),^28^ and the DSL rotation matrices were derived using the R package GPArotation (R Project for Statistical Computing).^29^

## Results

### Measurement Model in Parents

Of 2 947 703 individuals, 1 518 252 (51.5%) were male, and the mean (SD) age at the end of follow-up was 28.7 (8.9) years. The first 5 eigenvalues of the 10 parental conditions were 4.71, 1.33, 1.15, 0.65, and 0.58, so we decided to extract 3 exploratory factors. We rotated extracted factors toward a hierarchical model consisting of 1 general psychopathology factor (loading range, 0.43-0.66) and 3 specific psychotic, externalizing, and internalizing factors (Table). The factor-loading structure was highly similar for both fathers and mothers, suggesting that the hierarchical model was sex invariant (eMethods in Supplement 1).

**Table.** Factor Loadings and Model Fit of Exploratory Factor Analysis of 9 Parental Psychiatric Diagnoses and Violent Criminal Court Convictions^a^^,^^b^

Parental psychiatric diagnosis or criminality	Direct Schmid-Leiman rotation			
	General psychopathology factor	Specific factor		
		Psychotic	Externalizing	Internalizing
Bipolar disorder	0.62	0.42	−0.07	0.27
Schizophrenia	0.55	0.58	0.10	−0.13
Schizoaffective disorder	0.61	0.73	−0.08	−0.03
Alcohol-related disorders	0.58	−0.04	0.52	0.10
Drug-related disorders	0.60	−0.01	0.50	0.11
Violent crime convictions	0.44	0.00	0.44	0.00
Anxiety	0.60	0.00	0.13	0.47
Depression	0.66	0.07	0.02	0.57
Obsessive-compulsive disorder	0.43	0.05	−0.04	0.43
Posttraumatic stress disorder	0.55	−0.01	0.11	0.46

^a^ Factors standardized to a mean of 0 and a variance of 1.

^b^ Root mean square error of approximation = 0.012; confirmatory fit index = 0.997; Tucker-Lewis index = 0.992; χ^2^ = 7564.291; standardized root mean squared residual = 0.025; *P* < .001.

### Associations Between General Psychopathology Factor in Parents and Offspring Outcomes

The general psychopathology factor in parents was significantly associated with all 31 offspring outcomes (range: odds ratio [OR] for accidents, 1.08 [95% CI, 1.07-1.08] to OR for social welfare recipiency, 1.40 [95% CI, 1.39-1.40]) over and above the 3 specific factors and after false discovery rate multiple testing correction (eFigure 2 in Supplement 1). That is, children whose parents scored 1 SD above the mean on the general psychopathology factor had a statistically significant 8% to 40% higher odds of the outcomes. Decomposing all bivariate parent-offspring associations into attributable general vs specific psychopathology factors showed that they were mainly attributable to the general psychopathology factor (eFigure 3 in Supplement 1). Similarly, the general psychopathology factor in parents accounted for a large proportion of the explained variance in all offspring outcomes (eFigure 4 in Supplement 1).

### Associations Between Specific Psychopathology Factors In Parents and Offspring Outcome

The associations between specific parental psychopathology factors and offspring outcomes are shown in eFigure 2 in Supplement 1. Beyond the general psychopathology factor, the specific psychotic factor in parents was primarily and significantly associated with all 5 psychotic-like outcomes (range: OR for prescription of antiepileptics, 1.05 [95% CI, 1.04-1.06] to OR for schizophrenia, 1.25 [95% CI, 1.23-1.28]). Similarly, the specific internalizing factor in parents was primarily associated with all 6 internalizing outcomes (range: OR for prescription of anxiolytics, 1.10 [95% CI, 1.09-1.10] to OR for depression, 1.13 [95% CI, 1.12-1.13]) and all 6 neurodevelopmental outcomes (range: OR for intellectual disability, 1.02 [95% CI, 1.01-1.03] to OR for autism spectrum disorder, 1.10 [95% CI, 1.09-1.11]) in their offspring. The specific externalizing factor in parents was associated with the 6 externalizing outcomes (range: OR for violent crimes, 1.21 [95% CI, 1.19-1.23] to OR for oppositional defiant disorder, 1.32 [95% CI, 1.32-1.33]) as well as suicidal behavior (OR, 1.18 [95% CI, 1.17-1.19]), accidents (OR, 1.10 [95% CI, 1.10-1.11]), violent victimization (OR, 1.23 [95% CI, 1.22-1.24]), high school ineligibility (OR, 1.22 [95% CI, 1.21-1.23]), low school grade (OR, 1.28 [95% CI, 1.28-1.29]), low cognitive ability (OR, 1.12 [95% CI, 1.09-1.15]), social welfare recipiency (OR, 1.32 [95% CI, 1.31-1.32]), and unemployment (OR, 1.12 [95% CI, 1.11-1.12]) in offspring. In addition, the specific externalizing factor in parents was also associated with the 6 internalizing outcomes (range: OR for obsessive compulsive disorder, 1.01 [95% CI, 1.00-1.02] to OR for posttraumatic stress disorder, 1.13 [95% CI, 1.12-1.13]) and the 6 neurodevelopmental outcomes (range: OR for autism spectrum disorder, 1.04 [95% CI, 1.03-1.05] to OR for attention-deficit/hyperactivity disorder, 1.21 [95% CI, 1.20-1.22]). The pattern of associations for the general and specific psychopathology factors was largely similar for male and female offspring (eFigure 5 in Supplement 1).

These patterns were mirrored in terms of relative contributions. The bivariate correlations between parental psychotic diagnoses and offspring psychotic-like outcomes appeared to be partially attributable to the specific psychotic factor, in addition to the general psychopathology factor (eFigure 3A in Supplement 1). Similar patterns were also observed for the parental internalizing factor, which contributed to the correlations between parental internalizing diagnoses and offspring internalizing and neurodevelopmental outcomes (eFigure 3B in Supplement 1). In contrast, the externalizing factor accounted for the correlations between parental externalizing conditions and almost all offspring outcomes (eFigure 3C in Supplement 1). Similarly, when based on explained variance (eFigure 4 in Supplement 1), the specific psychotic and internalizing factors mainly explained the variance in psychotic-like outcomes and internalizing-related outcomes, respectively, while the externalizing factor explained the variance in almost all offspring outcomes.

### Sensitivity Analysis

The different hierarchical models in the sensitivity analyses all fit the data relatively well (eTable 3 in Supplement 1). The factor loadings are shown in eTable 4 in Supplement 1. All 3 sensitivity analyses generated similar patterns for the associations as the main analysis (eFigure 6 in Supplement 1). When only parents who had the condition prior to the offspring birth were examined, the associations between parent general psychopathology factor and most offspring outcomes were attenuated but remained statistically significant.

## Discussion

The general psychopathology factor in parents was significantly associated with all 31 outcomes in offspring and appeared to be primarily responsible for all the bivariate parent-offspring associations. Controlling for the general psychopathology factor, the specific psychotic and internalizing factors were primarily associated with within-spectrum and related offspring outcomes. In contrast, the specific externalizing factor was associated with both within-spectrum and across-spectra offspring outcomes to a greater extent.

### Associations Between the General Psychopathology Factor In Parents and Offspring Outcomes

Our findings replicate previous smaller children-of-twins studies that observed intergenerational transmission of general psychopathology factors^19,20^ and extend them to nonpsychiatric offspring outcomes and middle adulthood. Together, these findings suggest that psychiatric and behavioral-related parent-offspring associations might be attributable to a general vulnerability toward a broad range of mental health–related phenotypes. Using quantitative genetic modeling, the Swedish children-of-twins study suggested that this transmission might be attributed to direct phenotypic effects, passive gene-environment correlation (genes and environment are passed from parent-to-child generations), and/or nonpassive gene-environment correlation (parental psychopathology symptoms are evoked by children’s genetically influenced psychopathology symptoms, or children seek out environments consistent with their genetic predispositions).^19^ Regardless of the transmission pathway, professionals who work with children (eg, child psychologists, psychiatrists, teachers, and social workers) might benefit from taking the total number of parental psychiatric conditions into account, regardless of type, when forecasting child mental health and social functions. In addition, mental health professionals working with adult patients who have multiple psychiatric conditions might consider a more comprehensive screening and monitoring of their offspring.

### Associations Between Specific Psychopathology Factors In Parents and Offspring Outcomes

The specific psychotic factor in parents was primarily associated with psychotic-like outcomes in offspring, providing support for the specificity of the intergenerational transmission of psychotic disorders,^30,31,32^ above and beyond parental comorbidity. One possibility is that this transmission might be attributed to inherited genetics, as a Swedish adoption study showed that the intergenerational transmission of psychotic disorders was largely attributable to genetic factors.^33^ The lack of across-spectra associations suggests that previous findings of the transdiagnostic transmission from parental psychotic disorders to offspring nonpsychotic outcomes^34,35,36,37^ might be attributable to parental comorbidity.

Similar to the specific psychotic factor, the specific internalizing factor in parents was also primarily associated with within-spectrum and related outcomes (ie, neurodevelopmental outcomes) in offspring, dovetailing with previous findings of bivariate parent-offspring associations of internalizing disorder^10,38,39^ and an internalizing factor,^13^ even when controlling for parental comorbidity. As few across-spectra associations were found, our results corroborated the finding from the retrospective World Health Organization study that the within-spectrum associations were higher than the across-spectra associations.^2^ In contrast to the within-spectrum association found beyond the general psychopathology factor in our study, the Wisconsin Twin study found no transmission effects left for specific internalizing factor, as the transmission from parental internalizing factor to the offspring general psychopathology factor was sufficient in explaining the bivariate parent-offspring associations.^20^ This contradictory result may be attributable to the fact that the general psychopathology factor was derived from distinct individuals (parents vs offspring) and conditions were evaluated differently (by interviewers vs by specialists). Further research might be necessary to examine the intergenerational transmission of specific internalizing factors.

In contrast to the specific psychotic and internalizing factors, which were primarily associated with within-spectrum and related offspring outcomes, the specific externalizing factor in parents was associated with both within-spectrum (ie, externalizing outcomes) and some across-spectra outcomes (eg, internalizing outcomes and suicidal behavior) in offspring, which aligns with bivariate parent-offspring associations in the World Health Organization study^2^ and other previous research.^7,8,40,41^ This finding implies that a comprehensive evaluation might be beneficial when estimating the outcomes of children of parents who display criminality and harmful substance use. Regarding transmission pathways of the across-spectra associations, both shared genetic factors^7^ and environmental effects^42,43^ were identified in quantitative genetic studies. When controlling for both parental and offspring comorbidity, the associations between parental externalizing disorders and offspring internalizing outcomes disappeared,^10,13^ indicating that perhaps the across-spectra associations found in our study could potentially be attributed to offspring comorbidity.

### Limitations

Although our study relied on a large sample drawn from high-quality national registers and the follow-up time stretched up to 44 years of age in the offspring, several limitations should be acknowledged. First, the associations might be overestimated due to differential outcome misclassification^44^; that is, children of parents with several psychiatric conditions might be more prone to have psychiatric conditions themselves and receive care in the mental health system, such that they have a higher probability to be correctly identified than children whose parents have no or fewer psychiatric conditions. Second, the associations might not be generalizable to parents with psychiatric conditions who did not receive inpatient or outpatient treatment (ie, the results might not be generalizable to individuals treated only in primary care). Third, the current study was not designed to explore the mechanism of the intergenerational transmission of parental psychiatric comorbidity. Future studies could try to explore whether the associations between parental comorbidity and offspring outcomes were to remain after controlling for unmeasured confounders.

## Conclusions

In this cohort study of the Swedish population, we found that the intergenerational transmission of psychiatric conditions across different types of spectra appeared to be largely attributable to a general psychopathology factor, whereas specific psychopathology factors were primarily responsible for within-spectrum associations between parents and their offspring. Professionals who work with children (eg, child psychologists, psychiatrists, teachers, and social workers) might benefit from taking the total number of parental psychiatric conditions into account, regardless of type, when forecasting child mental health and social functions.

## Notes

Supplement 1. **eMethods.** Exam Whether the General Psychopathy Factor Model Was Sex Invariant **eTable 1.** Description of Registries and Variables Extracted **eTable 2.** The ICD/ATC Code, Classified Convictions for Violent Crimes, and the Cut-Off Age for Each Exposure and Outcome **eTable 3.** Model Fits for Measurement Models of Different Sensitivity Analyses **eTable 4.** Factor Loadings for Hierarchical Models of Different Sensitivity Analyses **eFigure 1.** An Exploratory Structural Equation Modeling Framework **eFigure 2.** Associations Between General and Specific Psychopathology Factors in Parents and Offspring Outcomes **eFigure 3.** Bivariate Parent-Offspring Correlations Decomposed Into General Versus Specific Psychopathology Factor Contributions **eFigure 4.** Proportion of Variance in the Outcomes Explained by Latent General and Specific Factors **eFigure 5.** Association Between General and Specific Psychopathology Factors in Parents and Offspring Outcomes, OR (95% CI), Plotting for Males and Females **eFigure 6.** Association Between General and Specific Psychopathology Factors in Parents and Offspring Outcomes, OR (95% CI), Plotting for All Analyses
eReferences 1.
**eAppendix.** Matrix Algebra, R, and Mplus Supplementary Code
eReferences 2.

Supplement 2.
Data Sharing Statement
