# Supplementary material for: Intergenerational Transmission of Psychiatric Conditions and Psychiatric, Behavioral, and Psychosocial Outcomes in Offspring
Source: JAMA Netw Open. 2023 Dec 20;6(12):e2348439. doi: 10.1001/jamanetworkopen.2023.48439 (PMC10733806; doi:10.1001/jamanetworkopen.2023.48439)
Supplement: Supplement 1. — eMethods. Exam Whether the General Psychopathy Factor Model Was Sex Invariant eTable 1. Description of Registries and Variables Extracted eTable 2. The ICD/ATC Code, Classified Convictions for Violent Crimes, and the Cut-Off Age for Each Exposure and Outcome eTable 3. Model Fits for Measurement Models of Different Sensitivity Analyses eTable 4. Factor Loadings for Hierarchical Models of Different Sensitivity Analyses eFigure 1. An Exploratory Structural Equation Modeling Framework eFigure 2. Associations Between General and Specific Psychopathology Factors in Parents and Offspring Outcomes eFigure 3. Bivariate Parent-Offspring Correlations Decomposed Into General Versus Specific Psychopathology Factor Contributions eFigure 4. Proportion of Variance in the Outcomes Explained by Latent General and Specific Factors eFigure 5. Association Between General and Specific Psychopathology Factors in Parents and Offspring Outcomes, OR (95% CI), Plotting for Males and Females eFigure 6. Association Between General and Specific Psychopathology Factors in Parents and Offspring Outcomes, OR (95% CI), Plotting for All Analyses eReferences 1. eAppendix. Matrix Algebra, R, and Mplus Supplementary Code eReferences 2. [file jamanetwopen-e2348439-s001.pdf]

Supplementary Online Content

Zhou M, Larsson H, D'Onofrio BM, Landén M, Lichtenstein P, Pettersson E. Intergenerational transmission of psychiatric conditions and psychiatric, behavioral, and psychosocial outcomes in offspring. *JAMA Netw Open.* 2023;6(12):e2348439. doi:10.1001/jamanetworkopen.2023.48439

eMethods. Exam Whether the General Psychopathy Factor Model Was Sex Invariant

eTable 1. Description of Registries and Variables Extracted

eTable 2. The ICD/ATC Code, Classified Convictions for Violent Crimes, and the Cut-Off Age for Each Exposure and Outcome

eTable 3. Model Fits for Measurement Models of Different Sensitivity Analyses

eTable 4. Factor Loadings for Hierarchical Models of Different Sensitivity Analyses

eFigure 1. An Exploratory Structural Equation Modeling Framework

eFigure 2. Associations Between General and Specific Psychopathology Factors in Parents and Offspring Outcomes

eFigure 3. Bivariate Parent-Offspring Correlations Decomposed Into General Versus Specific Psychopathology Factor Contributions

eFigure 4. Proportion of Variance in the Outcomes Explained by Latent General and Specific Factors

eFigure 5. Association Between General and Specific Psychopathology Factors in Parents and Offspring Outcomes, OR (95% CI), Plotting for Males and Females

eFigure 6. Association Between General and Specific Psychopathology Factors in Parents and Offspring Outcomes, OR (95% CI), Plotting for All Analyses

eReferences 1.

eAppendix. Matrix Algebra, R, and Mplus Supplementary Code

eReferences 2.

This supplementary material has been provided by the authors to give readers additional information about their work.

eMethods. Exam Whether the General Psychopathy Factor Model Was Sex Invariant

To examine whether the hierarchical factor model varied by sex, we constructed the model separately for fathers and mothers. We started out by examining the similarity of the factor loading structures using the factor congruence coefficient, which ranges from -1 to 1, with values above 0.95 indicating that the two factors can be considered equal(1). The factor congruence coefficients between the general factors for fathers and mothers were 1.00. The same result emerged for the three specific factors. Thus, the factor structure was highly similar for fathers and mothers, suggesting that the measurement model was sex invariant.

In addition, we also fitted two nested multiple group models to test the similarity of the factor loading structures between fathers and mothers. First, we allowed the loadings to vary between fathers and mothers. Second, we fit a more restrictive model where the loadings were forced to be the same for fathers and mothers. Although the more restrictive model fit significantly worse ($\Delta\chi2 = 1519.349$, df =26, $p < 0.001$), the absolute fit indices remained highly similar (ΔRMSEA = 0.001; ΔCFI = 0.000; ΔTLI = 0.001). Cheung and Rensvold recommended, based on simulations, that a ΔCFI <0.01 was inadequate to conclude that two nested models differed, suggesting that the factor structure was highly similar for mothers and fathers.(2)

eTable 1. Description of Registries and Variables Extracted

Register	Description	Variables
Total Population Register	Established in 1968 and includes demographic information (e.g., sex, age, place of birth) for the entire Swedish population.[1]	Individual identification number, birthyear
Multi-Generation Register	Links all index persons born in Sweden since 1932 and alive in 1960 to their biological parent.[2]	Individual identification number
National Patient Register	Includes individual-based records of psychiatric inpatient care since 1973 (complete since 1987) and psychiatric outpatient care since 2001 (complete since 2010). All diagnoses were recorded according to the International Classification of Diseases (ICD); 8th (1973/1969-1986), 9th (1987-1996), and 10th (1997-2009) revision.[3,4]	Disorders, suicide attempts, violent victimization, and accident
Prescribed Drug Register	Established in 2005 and contains all dispensed prescribed pharmaceuticals.[5]	Prescription of medication
National Crime Register	Comprises all registered criminal convictions of those aged 15 and older (the age of criminal responsibility) since 1973.[6]	Court convictions of violent crimes
National School Register	Includes averaged junior high final grades (age 15) and (in)eligibility for high school since 1988.	Low school grade, high school ineligibility
Cause of Death Register	Records all deaths in Sweden since 1952 and provides information on causes of death according to ICD.[7]	Death by suicide, death by accidents
Longitudinal Integration Database for Health Insurance and Market Studies	Information from the labor market and educational and social sectors for all individuals registered in Sweden over 16 years of age since 1990.[8]	Social welfare recipiency, unemployment, parental educational level
Conscription Register	Includes measures of general cognitive ability for 18-year-old males in stanine format (a nine-point scale with a mean of 5 and a standard deviation of 2).	Low cognitive ability

© 2023 Zhou M et al. *JAMA Network Open.*

eTable 2. The ICD/ATC Code, Classified Convictions for Violent Crimes, and the Cut-Off Age for Each Exposure and Outcome

Exposure/outcome	ICD 08 (1969-1986)	ICD 09 (1987-1996)	ICD 10 (1997-)	ATC code	Minimum age
Schizophrenia	295 (except 295.5 295.7)	295 (except 295F 295H)	F20		15
Schizoaffective disorder	295.7	295H	F25		15
Bipolar disorder	296.1, 296.3, 296.8, 296.9	296A, 296C, 296D, 296E, 296W, 296X	F30, F31		15
Depression	300.4	296B, 311, 300E	F32, F33		10
Anxiety	300 (except 300.3, 300.4)	300, (except 300E, 300D)	F40, F41		10
Obsessive-compulsive disorder	300.3	300D	F42		5
Post-traumatic stress disorder	307.99	308, 309	F43		2
Alcohol-related disorders	291, 303	291, 303, 305A	F10 (except F10.5)		12
Drug-related disorders	304	292, 304, 305X	F11-F19 (except F17, F1x.5)		12
Attention-Deficit/Hyperactivity Disorder	-	314	F90		3
Autism spectrum disorder	-	299A	F84.0, F84.1, F84.5		2
Tic disorder	306.2	307C	F95		3
Learning disorders	-	315A, 315B	F81		3
Intellectual disability	311-315	317-319	F70-F79		2
Oppositional defiant/conduct disorder	-	312X	F91		3
Suicide behavior (Suicide attempts and death by suicide)	E950-959 E980-989	E950-959 E980-989	X60-X84, Y10-Y34		10
Accidents (Injuries due to accidents/Death by accidents)	E807-E929	E807-E929	V01-V99, W00-W99, X00-X59		0
Violent victimization	E960 - E969	E960 - E969 (except E967)	X85-X99, Y01-Y09		0
Anti-alcohol medication				N07BB	12
Antidepressants				N06A	10
Antiepileptics				N03A	3
Anti-opioids medication				N07BC	12
Antipsychotic				N05AA-N05AL, N05AX	15
Anxiolytic				N05B	10
Lithium				N05AN	10
Stimulants				N06B	3
Violent crimes	homicide (Ch 3, §1-3); assault (Ch 3, §5-6); robbery (Ch 8, §5-6); threats and violence against an officer (Ch 17, §1-2); gross violation of a person's/woman's integrity (Ch 4, §4a); unlawful coercion (Ch 4, §4); unlawful threats (Ch 4, §5); kidnapping (Ch 4, §1); illegal confinement (Ch 4, §2);				15

© 2023 Zhou M et al. *JAMA Network Open.*

Exposure/outcome	ICD 08 (1969-1986)	ICD 09 (1987-1996)	ICD 10 (1997-)	ATC code	Minimum age
	arson (Ch 13, §1-2); intimidation (Ch 4, §7); sexual offence (excluding prostitution and the buying of sexual services but including child pornography) (Ch 6 §1-10, §10A, §12)				
High school ineligibility	Failure to achieve eligibility to advance to high school				15
Low school grade	Being ranked in the lowest quintile on the junior high school grade point average				15
Low cognitive ability	Defined as stanine 1, corresponding to the lowest 4%, only available for males				15
Unemployment	Have ever been unemployed				18
Social welfare recipiency	Have ever received the social welfare recipiency				18

© 2023 Zhou M et al. *JAMA Network Open.*

eTable 3. Model Fits for Measurement Models of Different Sensitivity Analyses

Measurement Models	*x*2	df	*p*	CFI	TLI	RMSEA	SRMR
Sensitivity 1a	21853.654	33	<0.001	0.990	0.987	0.015	0.052
Sensitivity 1b	7564.291	18	<0.001	0.997	0.992	0.012	0.025
Sensitivity 2a	0.000	0	<0.001	1.000	1.000	0.000	0.003
Sensitivity 2b	4244.224	7	<0.001	0.997	0.993	0.014	0.028
Sensitivity 3	277.130	18	<0.001	0.998	0.995	0.003	0.048

Note: CFI = comparative fit index; RMSEA = root mean square error of approximation; SRMR = standardized mean square residual; TLI = Tucker–Lewis Index. Sensitivity1a: a second-order CFA model; Sensitivity1b: a bifactor EFA model; Sensitivity2a: a second-order EFA model based on six parental diagnoses; Sensitivity2b: a second-order CFA model based on six parental diagnoses; Sensitivity3: a second-order EFA model, limited individuals whose parents were diagnosed before childbirth.

© 2023 Zhou M et al. *JAMA Network Open.*

eTable 4. Factor Loadings for Hierarchical Models of Different Sensitivity Analyses

	**Sensitivity 1a**				**Sensitivity 1b**				**Sensitivity 3**			
	General factor	Psychotic factor	Internalizing factor	Externalizing factor	General factor	Psychotic factor	Internalizing factor	Externalizing factor	General factor	Psychotic factor	Internalizing factor	Externalizing factor
**Based on ten parental diagnoses/criminality**												
Bipolar disorder	**0.63**	**0.66**			**0.76**	0.23		-0.09	**0.61**	**0.53**	0.16	-0.08
Schizophrenia	**0.50**	**0.52**			**0.56**	**0.58**		0.12	**0.61**	**0.52**	0.02	0.07
Schizoaffective disorder	**0.56**	**0.59**			**0.69**	**0.65**		-0.09	**0.64**	**0.53**	0.03	0.07
Alcohol-related disorders	**0.55**			**0.57**	**0.52**	-0.03		**0.59**	**0.63**	-0.10	0.14	**0.59**
Drug-related disorders	**0.57**			**0.58**	**0.55**	-0.02		**0.56**	**0.65**	0.03	0.00	**0.61**
Violent crime	**0.40**			**0.41**	**0.36**	0.04		**0.50**	**0.39**	0.02	-0.13	**0.50**
Anxiety	**0.79**				**0.72**	-0.23		0.12	**0.60**	0.03	**0.39**	0.17
Depression	**0.85**				**0.85**	-0.23		0.00	**0.67**	0.05	**0.48**	0.13
Obsessive-compulsive disorder	**0.58**				**0.58**	-0.19		-0.07	**0.49**	0.13	**0.42**	-0.06
Post-traumatic stress disorder	**0.73**				**0.68**	-0.23		0.10	**0.55**	0.10	0.23	0.22

	**Sensitivity 2a**				**Sensitivity 2b**			
	General factor	Psychotic factor	Internalizing factor	Externalizing factor	General factor	Psychotic factor	Internalizing factor	Externalizing factor
**Based on six parental diagnoses**								
Bipolar disorder	**0.65**	**0.58**	0.16	-0.09	**0.67**	**0.52**		
Schizophrenia	**0.45**	**0.36**	-0.01	0.11	**0.45**	**0.36**		
Alcohol-related disorders	**0.56**	0.01	0.13	**0.43**	**0.56**			**0.53**
Drug-related disorders	**0.65**	0.01	0.02	**0.62**	**0.59**			**0.56**
Anxiety	**0.60**	0.06	**0.41**	0.14	**0.79**			
Depression	**0.69**	0.10	**0.59**	0.01	**0.84**			

Note: Sensitivity1a: a second-order CFA model; Sensitivity1b: a bifactor EFA model; Sensitivity2a: a second-order EFA model based on six parental diagnoses; Sensitivity2b: a second-order CFA model based on six parental diagnoses; Sensitivity3: a second-order EFA model, limited individuals whose parents were diagnosed before childbirth.

© 2023 Zhou M et al. *JAMA Network Open.*

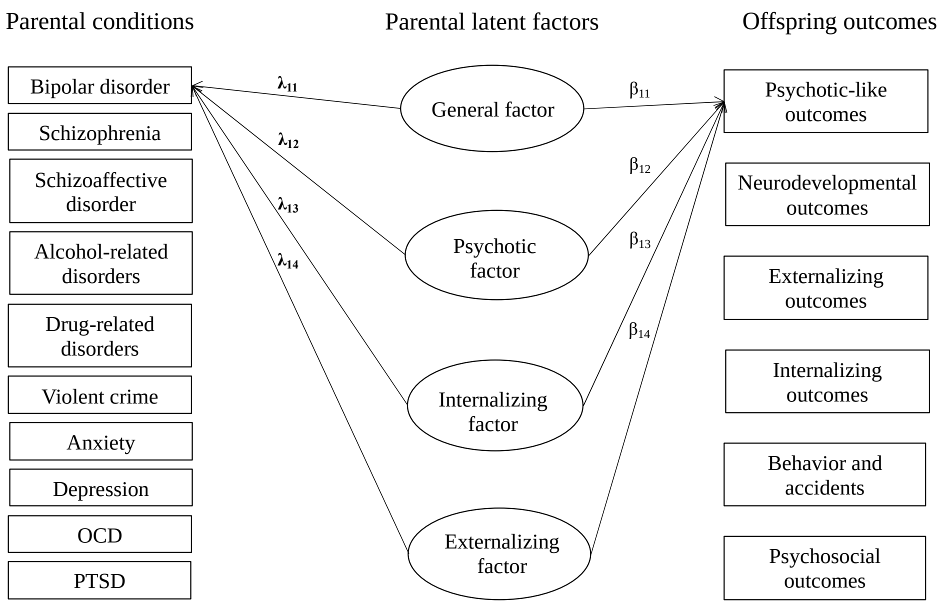

eFigure 1. An Exploratory Structural Equation Modeling Framework

Note: All other arrows among parental exposures, parental latent factor, and offspring outcomes were omitted for visualization. Offspring outcomes were listed as clusters. 1) λ_{11}* β_{11} is the bivariate correlation between parental bipolar disorder and offspring psychotic outcomes (e.g. offspring bipolar disorder) that could be attributable to general psychopathology factor. 2) β_{11}^2/(β_{11}^2+ β_{12}^2+ β_{13}^2+ β_{14}^2) is the proportion of variance in offspring psychotic outcomes (e.g. offspring bipolar disorder) that could be explained by the general psychopathology factor. Abbreviations: OCD = obsessive-compulsive disorder, PTSD = post-traumatic stress disorder

© 2023 Zhou M et al. *JAMA Network Open.*

Offspring Outcome	General factor OR (95% CI)	Specific psychotic factor OR (95% CI)	Specific internalizing factor OR (95% CI)	Specific externalizing factor OR (95% CI)
**Psychotic-like outcome**				
Bipolar disorder	1.28 (1.27-1.28)	1.20 (1.19-1.22)	1.05 (1.04-1.06)	1.01 (1.00-1.02)
Lithium	1.25 (1.24-1.26)	1.24 (1.22-1.26)	1.04 (1.03-1.05)	0.97 (0.96-0.98)
Antiepileptics	1.20 (1.20-1.21)	1.05 (1.04-1.06)	1.05 (1.04-1.05)	1.09 (1.09-1.10)
Schizophrenia	1.23 (1.22-1.24)	1.25 (1.23-1.28)	0.96 (0.94-0.97)	1.03 (1.01-1.04)
Antipsychotic	1.28 (1.27-1.29)	1.11 (1.10-1.13)	1.04 (1.03-1.05)	1.10 (1.10-1.11)
**Neurodevelopmental outcomes**				
ADHD	1.29 (1.28-1.29)	0.99 (0.98-1.00)[b]	1.08 (1.07-1.09)	1.21 (1.20-1.22)
Stimulants	1.27 (1.26-1.27)	0.98 (0.97-0.99)	1.08 (1.07-1.09)	1.20 (1.20-1.21)
Tic disorder	1.16 (1.14-1.17)	1.01 (0.99-1.04)[b]	1.08 (1.06-1.10)	1.06 (1.04-1.08)
ASD	1.18 (1.17-1.19)	1.04 (1.02-1.05)	1.10 (1.09-1.11)	1.04 (1.03-1.05)
Intellectual disability	1.17 (1.16-1.18)	1.06 (1.04-1.07)	1.02 (1.01-1.03)	1.09 (1.08-1.10)
Learning disorders	1.13 (1.12-1.14)	0.99 (0.97-1.01)[b]	1.05 (1.04-1.07)	1.08 (1.06-1.09)
**Internalizing outcomes**				
Anxiety	1.25 (1.25-1.26)	1.00 (0.99-1.01)[b]	1.12 (1.11-1.12)	1.12 (1.11-1.12)
Anxiolytic	1.21 (1.21-1.21)	1.00 (0.99-1.00)[b]	1.10 (1.09-1.10)	1.11 (1.11-1.12)
Depression	1.24 (1.24-1.25)	1.02 (1.01-1.03)	1.13 (1.12-1.13)	1.08 (1.08-1.09)
Antidepressants	1.22 (1.21-1.22)	1.00 (0.99-1.01)[b]	1.11 (1.11-1.11)	1.10 (1.09-1.10)
PTSD	1.24 (1.24-1.25)	0.99 (0.98-1.00)[b]	1.11 (1.11-1.12)	1.13 (1.12-1.13)
OCD	1.15 (1.14-1.16)	1.02 (1.00-1.04)	1.11 (1.10-1.13)	1.01 (1.00-1.02)[b]
**Externalizing outcomes**				
Alcohol-related disorders	1.25 (1.24-1.26)	0.99 (0.97-1.00)	1.00 (0.99-1.01)[b]	1.27 (1.26-1.28)
Anti-alcohol medication	1.24 (1.24-1.25)	0.97 (0.96-0.99)	1.02 (1.01-1.03)	1.26 (1.25-1.27)
Drug-related disorders	1.31 (1.31-1.32)	0.99 (0.97-1.00)	1.02 (1.02-1.03)	1.30 (1.29-1.31)
Anti-opioids medication	1.24 (1.22-1.25)	0.98 (0.95-1.01)[b]	0.99 (0.96-1.01)[b]	1.28 (1.26-1.30)
ODD	1.28 (1.27-1.29)	0.99 (0.97-1.01)[b]	0.99 (0.98-0.99)	1.32 (1.32-1.33)
Violent crimes	1.26 (1.26-1.27)	0.97 (0.96-0.98)	1.07 (1.05-1.08)	1.21 (1.19-1.23)
**Psychosocial outcomes**[a]				
High school ineligibility	1.22 (1.21-1.23)	0.95 (0.94-0.96)	1.05 (1.05-1.06)	1.22 (1.21-1.23)
Low school grade	1.23 (1.23-1.23)	0.93 (0.93-0.94)	1.03 (1.02-1.03)	1.28 (1.28-1.29)
Social welfare recipiency	1.40 (1.39-1.40)	0.97 (0.96-0.98)	1.09 (1.09-1.10)	1.32 (1.31-1.32)
Unemployment	1.12 (1.11-1.12)	0.98 (0.97-0.99)	1.02 (1.02-1.03)	1.12 (1.11-1.12)
Low cognitive ability[c]	1.11 (1.09-1.13)	0.99 (0.95-1.03)[b]	1.00 (0.98-1.03)[b]	1.12 (1.09-1.15)
**Behavior and accidents**				
Accidents	1.08 (1.07-1.08)	0.97 (0.96-0.97)	1.01 (1.01-1.01)	1.10 (1.10-1.11)
Suicide behavior	1.22 (1.22-1.23)	0.99 (0.98-1.00)[b]	1.04 (1.04-1.05)	1.18 (1.17-1.19)
Violent victimization	1.20 (1.19-1.20)	0.97 (0.96-0.98)	1.01 (1.00-1.02)	1.23 (1.22-1.24)

eFigure 2. Associations Between General and Specific Psychopathology Factors in Parents and Offspring Outcomes

Note: [a] Sample sizes varied for high school ineligibility (1 546 888 individuals), low school grade (2 398 752 individuals), social welfare recipiency and unemployment (2 497 731 individuals), low cognitive ability (156 239 individuals) depending on outcome missing information. [b] Odds ratios do not meet criteria for false discovery rate statistical significance [c] only for male. Abbreviations: ADHD = Attention-Deficit/Hyperactivity Disorder, ASD = autism spectrum disorder, PTSD = post-traumatic stress disorder, OCD = obsessive-compulsive disorder, ODD = oppositional defiant disorder.

© 2023 Zhou M et al. *JAMA Network Open.*

A

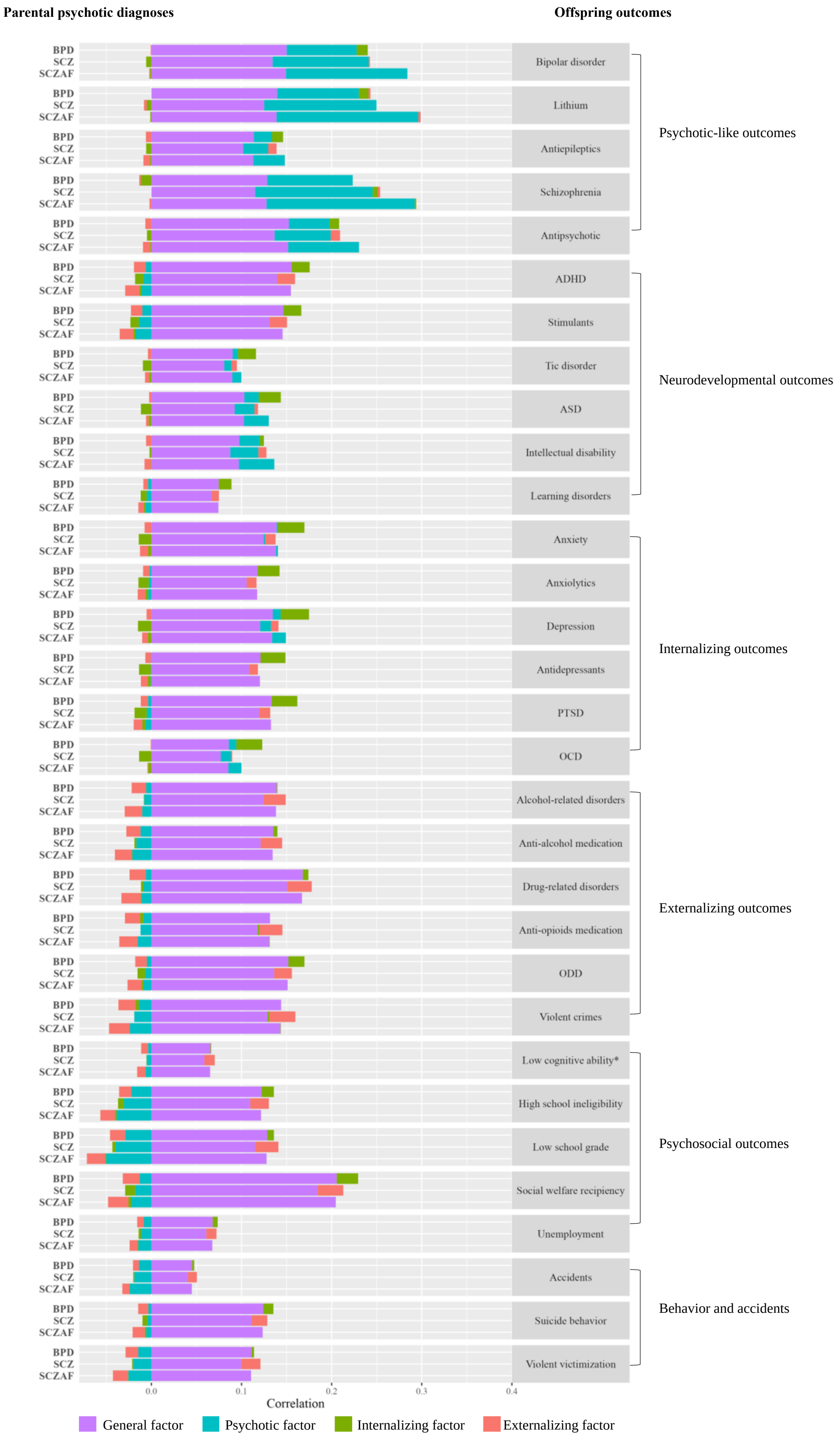

© 2023 Zhou M et al. *JAMA Network Open.*

B

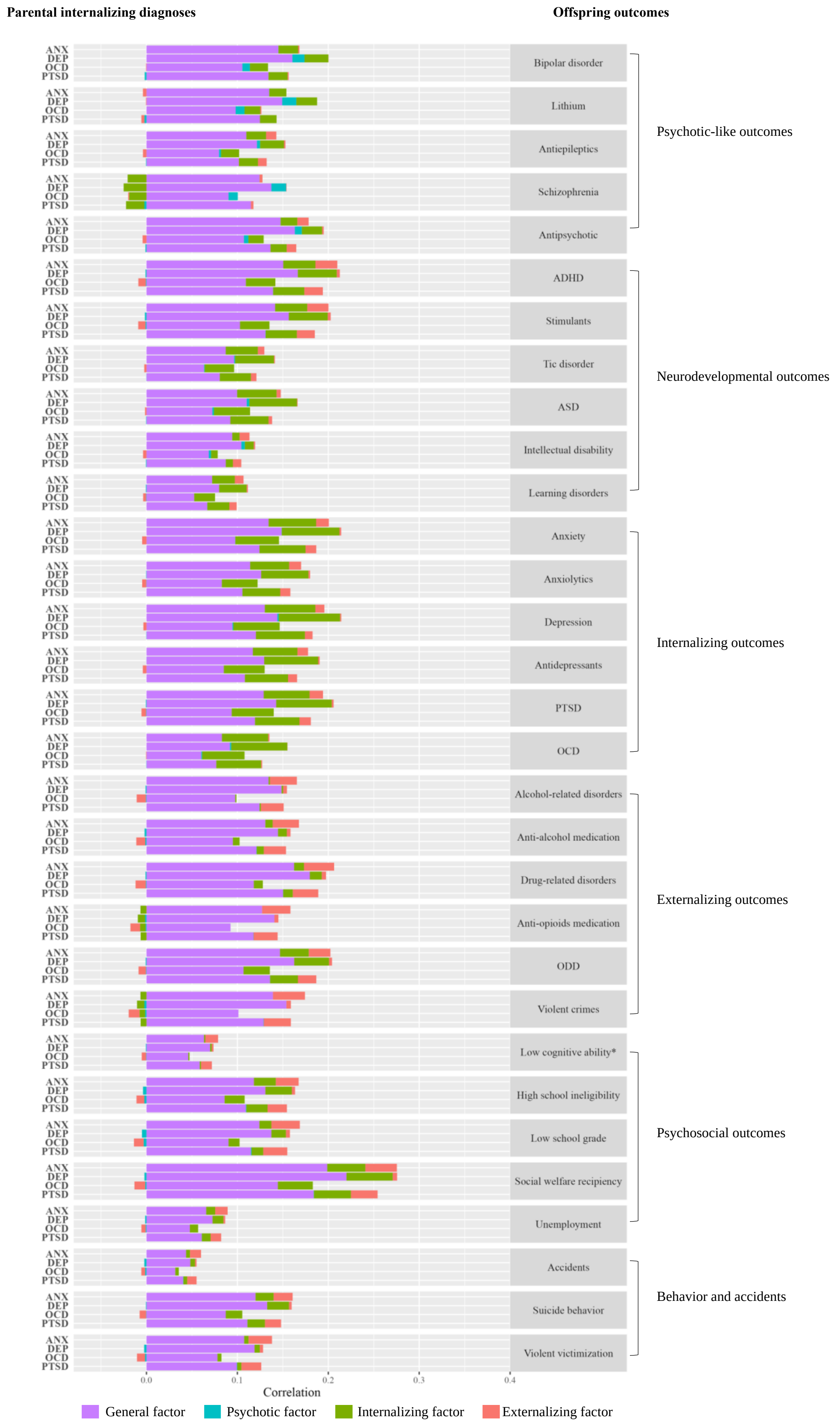

© 2023 Zhou M et al. *JAMA Network Open.*

C

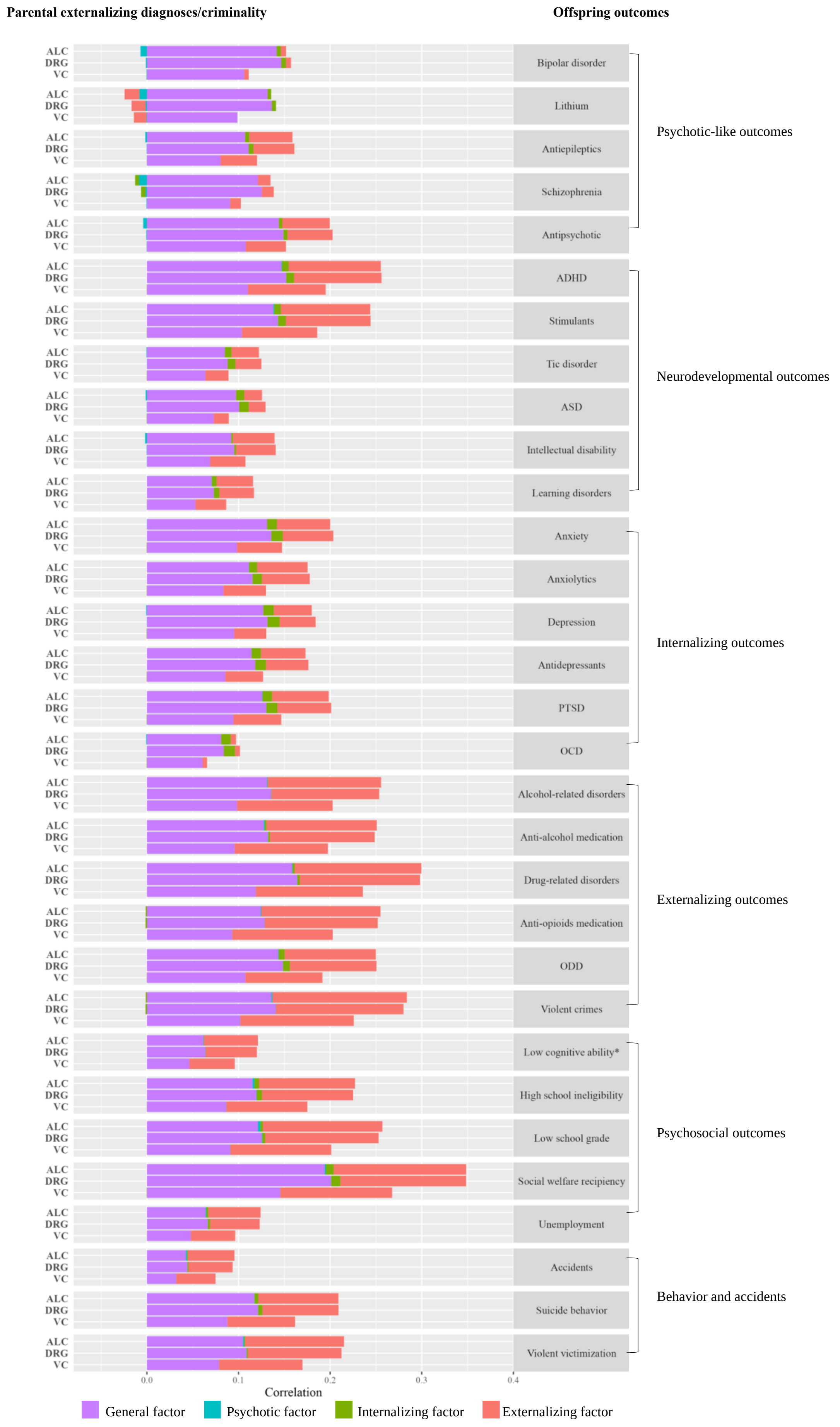

eFigure 3. Bivariate Parent-Offspring Correlations Decomposed Into General Versus Specific Psychopathology Factor Contributions

Note: Different colors represent the estimated correlation that can be attributed to the latent factor. Abbreviations: BPD = bipolar disorder, SCZ = schizophrenia, SCZAF = schizoaffective disorder, ANX = anxiety, DEP = depression, OCD = obsessive-compulsive disorder, PTSD = post-traumatic stress disorder, ALC =

© 2023 Zhou M et al. *JAMA Network Open.*

alcohol-related disorders, DRG = drug-related disorders, VC = violent crimes, ADHD = Attention-Deficit/Hyperactivity Disorder, ASD = autism spectrum disorder, ODD = oppositional defiant disorder.

© 2023 Zhou M et al. *JAMA Network Open.*

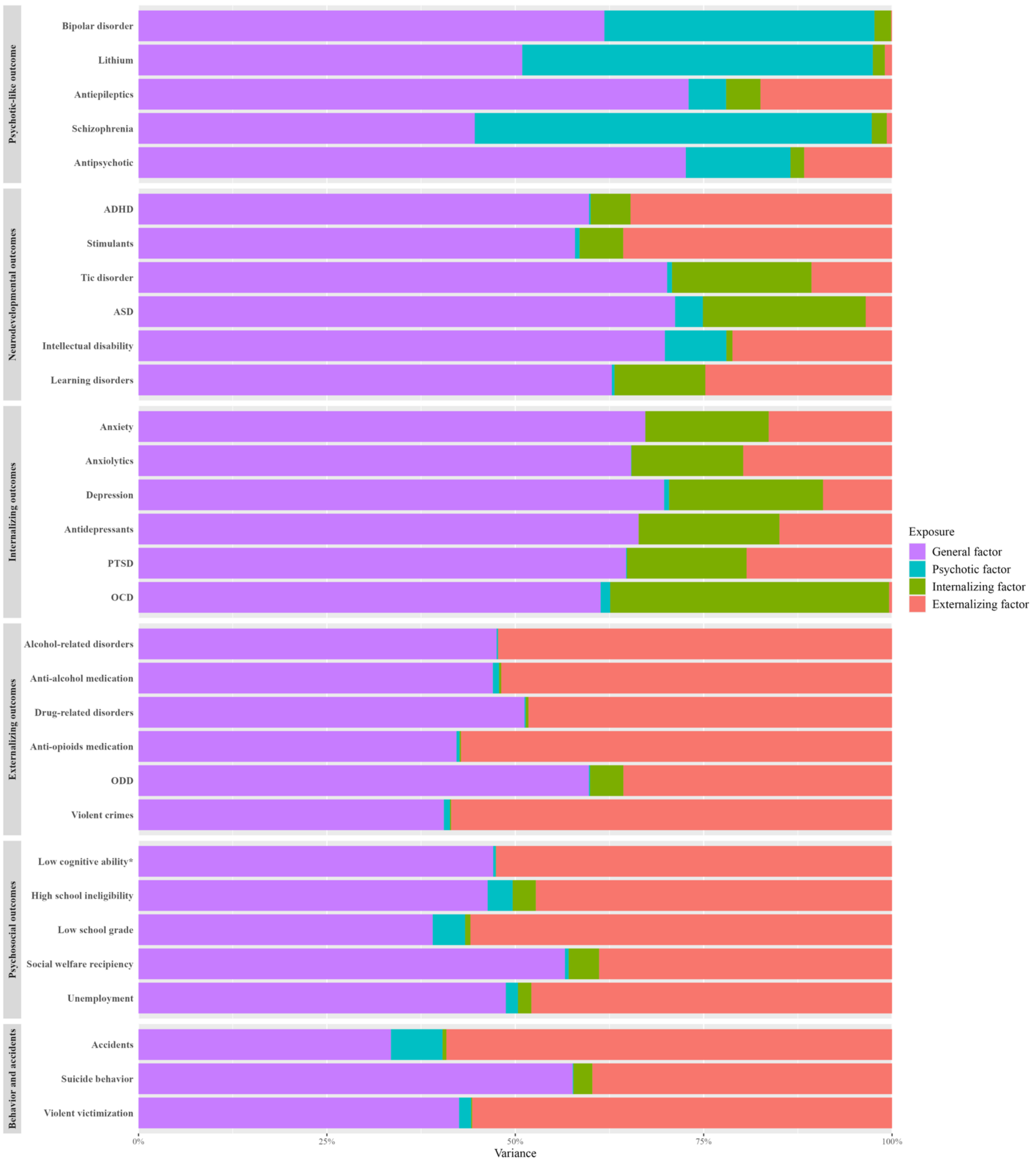

eFigure 4. Proportion of Variance in the Outcomes Explained by Latent General and Specific Factors
Note: Abbreviations: ADHD= Attention-Deficit/Hyperactivity Disorder, ASD= autism spectrum disorder, PTSD= post-traumatic stress disorder, OCD= obsessive-compulsive disorder, ODD= oppositional defiant disorder. *: only for male.

© 2023 Zhou M et al. *JAMA Network Open.*

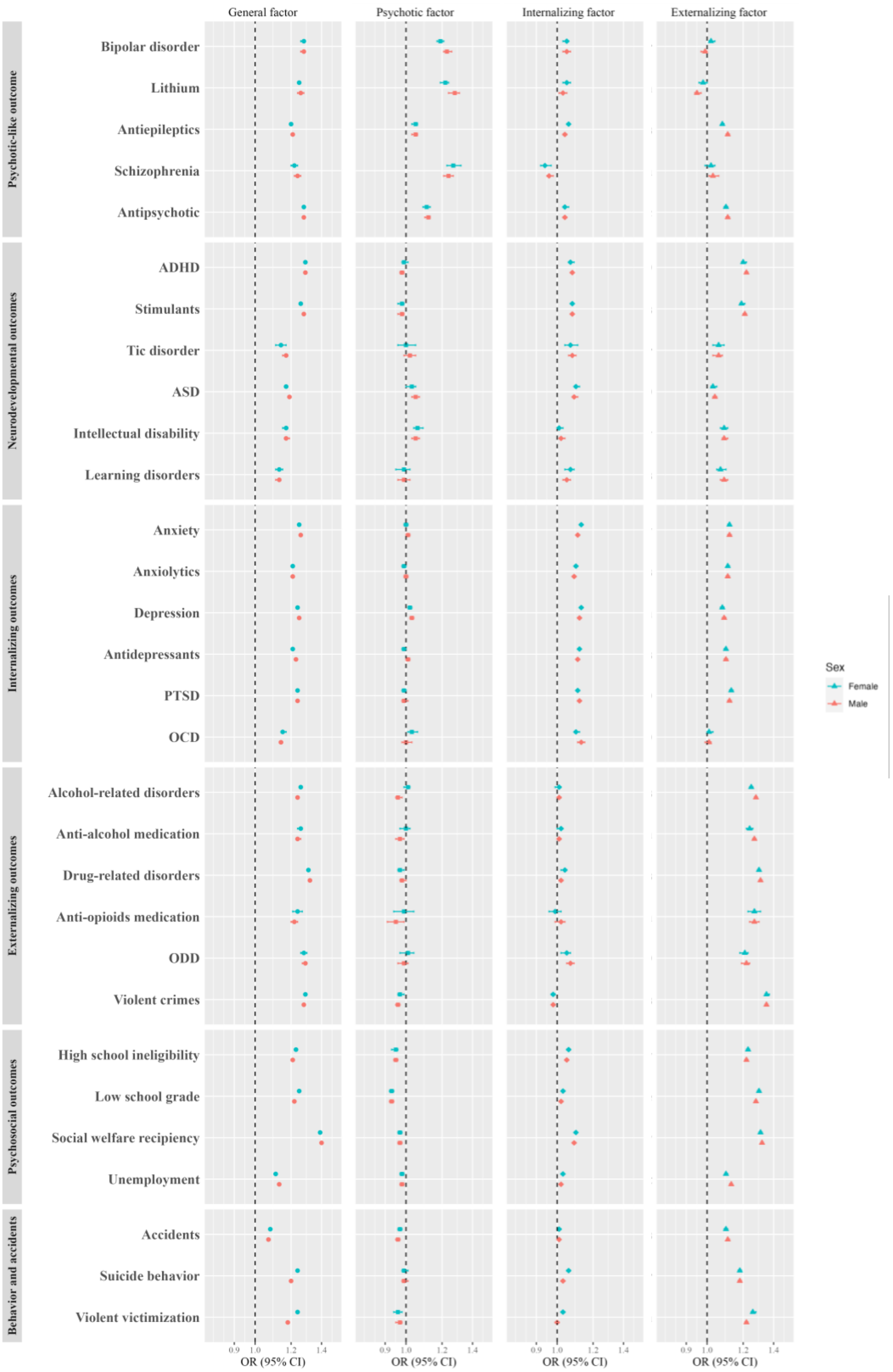

eFigure 5. Association Between General and Specific Psychopathology Factors in Parents and Offspring Outcomes, OR (95% CI), Plotting for Males and Females

Note: Abbreviations: ADHD= Attention-Deficit/Hyperactivity Disorder, ASD= autism spectrum disorder, PTSD= post-traumatic stress disorder, OCD= obsessive-compulsive disorder, ODD= oppositional defiant disorder. The association for low cognitive ability was not shown as it was only measured for males (see eFigure 6).

© 2023 Zhou M et al. *JAMA Network Open.*

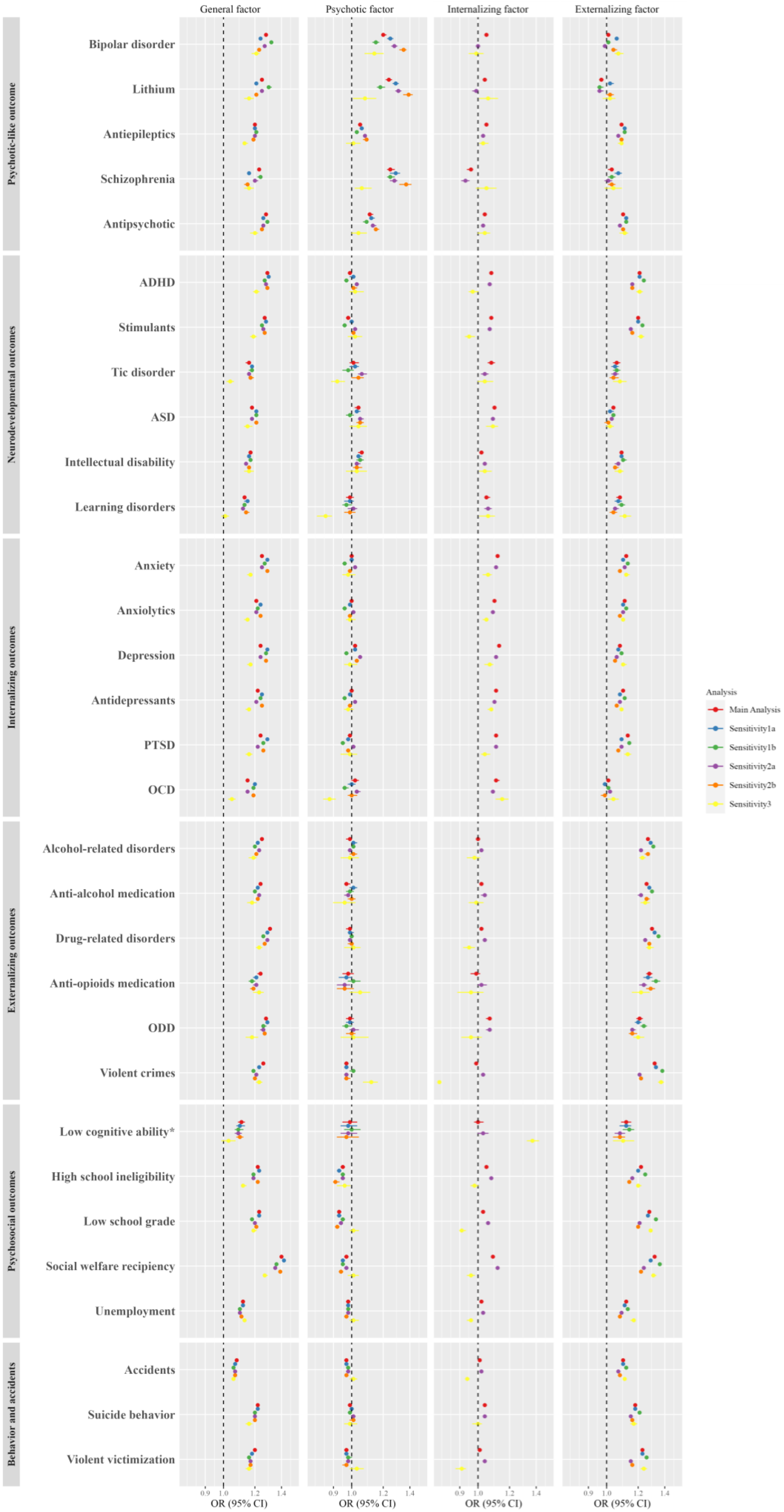

© 2023 Zhou M et al. *JAMA Network Open.*

eFigure 6. Association Between General and Specific Psychopathology Factors in Parents and Offspring Outcomes, OR (95% CI), Plotting for All Analyses

Note: Sensitivity1a: a second-order CFA model; Sensitivity1b: a bifactor EFA model; Sensitivity2a: a second-order EFA model based on six parental diagnoses; Sensitivity2b: a second-order CFA model based on six parental diagnoses; Sensitivity3: a second-order EFA model, limited individuals whose parents were diagnosed before childbirth. Abbreviations: ADHD= Attention-Deficit/Hyperactivity Disorder, ASD= autism spectrum disorder, PTSD= post-traumatic stress disorder, OCD= obsessive-compulsive disorder, ODD= oppositional defiant disorder. *: only for male.

© 2023 Zhou M et al. *JAMA Network Open.*

eReferences.

1. Ludvigsson JF, Almqvist C, Bonamy A-KE, et al. Registers of the Swedish total population and their use in medical research. European Journal of Epidemiology 2016;31(2):125-136. DOI: 10.1007/s10654-016-0117-y.
2. Ekbom A. The Swedish Multi-generation Register. Methods in Molecular Biology: Humana Press; 2011:215-220.
3. Socialstyrelsen. The National Patient Register. (https://www.socialstyrelsen.se/en/statistics-and-data/registers/national-patient-register/).
4. Ludvigsson JF, Andersson E, Ekbom A, et al. External review and validation of the Swedish national inpatient register. BMC Public Health 2011;11(1):450. DOI: 10.1186/1471-2458-11-450.
5. Wettermark B, Hammar N, Michaelfored C, et al. The new Swedish Prescribed Drug Register—Opportunities for pharmacoepidemiological research and experience from the first six months. Pharmacoepidemiology and Drug Safety 2007;16(7):726-735. DOI: 10.1002/pds.1294.
6. Frisell T, Lichtenstein P, Långström N. Violent crime runs in families: a total population study of 12.5 million individuals. Psychological Medicine 2011;41(1):97-105. DOI: 10.1017/s0033291710000462.
7. Brooke HL, Talbäck M, Hörnblad J, et al. The Swedish cause of death register. European Journal of Epidemiology 2017;32(9):765-773. DOI: 10.1007/s10654-017-0316-1.
8. Ludvigsson JF, Svedberg P, Olén O, Bruze G, Neovius M. The longitudinal integrated database for health insurance and labour market studies (LISA) and its use in medical research. European Journal of Epidemiology 2019;34(4):423-437. DOI: 10.1007/s10654-019-00511-8.

© 2023 Zhou M et al. *JAMA Network Open.*

eAppendix. Matrix Algebra, R, and Mplus Supplementary Code

1. Applying a Direct Schmid-Leiman rotation manually within an ESEM framework: Matrix algebra
2. Applying a Direct Schmid-Leiman rotation manually within an ESEM framework: Mplus- and R-code
3. Applying a second-order CFA measurement model

These appendices address an issue with second-order models, namely, that they lack one degree of freedom when estimating associations between a latent measurement model and exposures/outcomes. Specifically, because second-order models estimate x factors, they can only estimate x associations with outcomes. Second-order models, however, generate x+1 factors, such that they lack one degree of freedom when estimating associations with exposures/outcomes. One approach to circumvent this issue is to only estimate x associations with the exposures/outcomes. For example, one could constrain one specific factor to have zero residual and therefore it would have no association with the exposures/outcomes. Alternatively, one could constrain two associations to equality such that one only estimates x associations with the exposures/outcomes (this might be defensible if two specific factors capture similar constructs). Yet another approach is to use a Direct Schmid-Leiman (DSL) rotation, which simply re-distributes the x associations into x+1 associations. We used the first approach in the second-order CFA model and the latter approach in the second-order EFA model.

1. Applying a Direct Schmid-Leiman rotation manually within an ESEM framework: Matrix algebra

To circumvent the issue with second-order EFA model lacking one degree of freedom when estimating associations with outcomes, we applied a DSL rotation within an Exploratory Structural Equation Modeling (ESEM) framework.

This DSL rotation approximates a so-called second-order hierarchical factor model, which has previously been identified in three steps. First, a set of lower-order factors are extracted (e.g., based on EFA). Second, a higher-order factor is in turn extracted based on the correlations among the lower-order factors, such that the lower-order factors are decomposed into a part that is explained by the higher-order factor and a residual part that is not. Third, the second-order model is converted into a hierarchical model by computing the associations between the higher-order and the residual parts of the lower-order factors, and the observed indicators. In contrast to prior hierarchical factor models, the DSL rotation achieves this three-step process with a single rotation. The higher-order factor is usually labeled a general factor, and the residual lower-order factors are usually labeled specific factors.

The software Mplus (https://www.statmodel.com/) contains various rotations that can be used within an ESEM framework. However, it does not (yet) include the DSL rotation. Below we outline the basic ESEM equations as applied to the lambda (factor loading pattern) and beta (regressions between factors and exposures/outcomes) matrices and the rotation of beta matrix, which might be helpful to review before reading the practical steps of how to apply this rotation manually below. To simplify the estimation, Mplus treats all outcomes as latent variables (that are perfectly indicated by their observed counter-part). Therefore, we applied the rotation to the beta matrix containing the regression of the (latent) outcome onto the latent measurement model.

ESEM equations:

$$x = \tau_x + \Lambda_x \eta + \delta$$
$$\eta = \alpha + \mathrm{B}\,\eta + \mathrm{K}z + \zeta$$

$$\begin{bmatrix}\eta_1\\ \eta_2\\ \eta_3\\ \mathrm{y}\end{bmatrix} = \begin{bmatrix}a_1\\ a_2\\ a_3\\ a_4\end{bmatrix} + \begin{bmatrix}0 & 0 & 0 & 0\\ 0 & 0 & 0 & 0\\ 0 & 0 & 0 & 0\\ \beta_{41} & \beta_{42} & \beta_{43} & 0\end{bmatrix}\begin{bmatrix}\eta_1\\ \eta_2\\ \eta_3\\ \mathrm{y}\end{bmatrix} + \begin{bmatrix}0 & 0\\ 0 & 0\\ 0 & 0\\ k_{41} & k_{42}\end{bmatrix}\begin{bmatrix}z_1\\ z_2\end{bmatrix} + \begin{bmatrix}\zeta_1\\ \zeta_2\\ \zeta_3\\ \zeta_4\end{bmatrix}$$

Rotation:

$$\begin{aligned}\eta_{rot} &= \mathrm{H}\,\eta\\ &= \mathrm{H}\,(\alpha + \mathrm{B}\,\eta + Kz + \zeta)\\ &= \mathrm{H}\,\alpha + \mathrm{H}\,\mathrm{B}\,\mathrm{H}^{-1}\,\mathrm{H}\,\eta + Kz + \mathrm{H}\,\zeta\\ &= \mathrm{H}\,\alpha + \mathrm{H}\,\mathrm{B}\,\mathrm{H}^{-1}\,\eta_{rot} + \mathrm{H}\,Kz + \mathrm{H}\,\zeta\ (\eta_{rot} = \mathrm{H}\,\eta)\end{aligned}$$

$$\mathrm{B}^* = \mathrm{H}\,\mathrm{B}\,\mathrm{H}^{-1}$$

x is the indicator (parental exposures), η is the latent factor (offspring outcomes were also treated as latent factor in Mplus), z is the covariate variable (i.e., offspring birth year, the highest parental educational level), Λ_x is the factor loading matrix, B is the beta matrix of the latent factor on the latent factor (also offspring outcomes), K is the beta matrix of the covariate on the outcomes, τ_x and α are intercepts, δ and ζ are the error terms. H is the rotation matrix, B^* is the rotated beta matrix.

© 2023 Zhou M et al. *JAMA Network Open.*

2. Applying a Direct Schmid-Leiman rotation manually within an ESEM framework: Mplus- and R-code

Step 1: Derive echelon pattern matrix in Mplus based on ten parental psychiatric conditions

```
TITLE:
        202306_efa;
DATA:
        FILE = "mgr_mz_psy_use";
VARIABLE:
        NAMES = bpdfm depfm drgfm anxfm alcfm sczfm Sczaffectfm ocdfm ptsdfm ccfm;
        MISSING=.;
        USEVARIABLES = bpdfm sczfm Sczaffectfm alcfm drgfm ccfm anxfm depfm ocdfm ptsdfm;
        CATEGORICAL = bpdfm sczfm Sczaffectfm alcfm drgfm ccfm anxfm depfm ocdfm ptsdfm;
ANALYSIS:
        MODEL = nocovariances;
        PROCESS = 4;
        ESTIMATOR = wlsmv;
MODEL:
        F1 by bpdfm-ptsdfm*(Afix1 - Afix10);
        F2 by bpdfm-ptsdfm*(Bfix1 - Bfix10);
        F3 by bpdfm-ptsdfm*(Cfix1 - Cfix10);
        F1- F3@1;

MODEL CONSTRAINT:
        !FIXING FACTOR LOADINGS TO ECHELON PATTERN
        Afix3 < 0.99; !Afix3 would be larger than 1 if not constrained
        Bfix3 = 0;
        Cfix2 = 0;
        Cfix3 = 0;

OUTPUT:
STAND; STANDARDIZED; TECH1; TECH4; SAMPSTAT;
```

Step 2: Derive the Direct Schmid-Leiman rotation in R, based on above echelon pattern matrix

Step 2a. Read in factor loadings from step 1

```
test <- matrix(
         c(
          0.698,0.760,0.990,0.285,0.324,0.234,0.335,0.435,0.277,0.301,
          0.008,0.197,0.000,0.652,0.622,0.544,0.205,0.093,0.001,0.177,
          0.376,0.000,0.000,0.347,0.361,0.189,0.664,0.756,0.551,0.634),
          nrow=10,ncol=3)
rownames(test) <- c("bpdfm","sczfm","Sczaffectfm","alcfm","drgfm","ccfm","anxfm","depfm","ocdfm","ptsdfm")
```

Step 2b. Do the DSL rotation and get the rotation matrix

```
test2.dirSL <- targetT(
                cbind(test,0),
                Target = cbind(1,factor2cluster(Varimax(test)$loadings, cut = 0)))
rot <- t(test2.dirSL$Th)  ##derive the DSL rotation matrix
```

```
> rot #rot is rotation matrix H
  [,1]       [,2]       [,3]       [,4]
[1,]  0.62575015  0.3709949  0.46989273 -0.5000003
[2,]  0.77287183 -0.2262035 -0.31859177  0.5000004
[3,] -0.08546519  0.8611275 -0.03404132  0.4999963
[4,] -0.06165699 -0.2639208  0.82252130  0.5000029
```

Step 3: Do the ESEM in the Mplus software with the rotation matrix applied in scalar form (as MPlus does not allow for matrix algebra) in the Model Constraint command.

```
TITLE:
        202306_efa_esem;
DATA:
        FILE = "mgr_mz_psy_use";
VARIABLE:
        NAMES = bpdfm depfm drgfm anxfm alcfm sczfm Sczaffectfm ocdfm ptsdfm ccfm
```

© 2023 Zhou M et al. *JAMA Network Open.*

```
                Outcome  BirYearO  eduP lopnrM;
        MISSING=.;
        USEVARIABLES = bpdfm sczfm Sczaffectfm alcfm drgfm ccfm anxfm depfm ocdfm
                       ptsdfm outcome  BirYearO  eduP lopnrM;
        CATEGORICAL = bpdfm sczfm Sczaffectfm alcfm drgfm ccfm anxfm depfm ocdfm ptsdfm
                      outcome;
        CLUSTER is lopnrM;
DEFINE:
        STANDARDIZE BirYearO ;
ANALYSIS:
        MODEL = nocovariances;
        TYPE = COMPLEX;
        PROCESS = 4;
        ESTIMATOR = wlsmv;
MODEL:
        F1 by bpdfm-ptsdfm*(Afix1 - Afix10);
        F2 by bpdfm-ptsdfm*(Bfix1 - Bfix10);
        F3 by bpdfm-ptsdfm*(Cfix1 - Cfix10);
        F1- F3@1;

        !REGRESSIONS
        outcome on F1 (b1);
        outcome on F2 (b2);
        outcome on F3 (b3);
        outcome on BirYearO (b4);
        outcome on eduP (b5);

        BirYearO with F1 (r41);
        BirYearO with F2 (r42);
        BirYearO with F3 (r43);

        eduP with F1 (r51);
        eduP with F2 (r52);
        eduP with F3 (r53);
MODEL CONSTRAINT:
        !FIXING FACTOR LOADINGS TO ECHELON PATTERN (DERIVE FROM STEP 1)
         Afix1 = 0.698;
         Afix2 = 0.760;
         Afix3 = 0.990;
         Afix4 = 0.285;
         Afix5 = 0.324;
         Afix6 = 0.234;
         Afix7 = 0.335;
         Afix8 = 0.435;
         Afix9 = 0.277;
         Afix10 =0.301;

         Bfix1 = 0.008;
         Bfix2 = 0.197;
         Bfix3 = 0;
         Bfix4 = 0.652;
         Bfix5 = 0.622;
         Bfix6 = 0.544;
         Bfix7 = 0.205;
         Bfix8 = 0.093;
         Bfix9 = 0.001;
         Bfix10 = 0.177;

         Cfix1 = 0.376;
         Cfix2 = 0;
         Cfix3 = 0;
         Cfix4 = 0.347;
         Cfix5 = 0.361;
         Cfix6 = 0.189;
         Cfix7 = 0.664;
```

© 2023 Zhou M et al. *JAMA Network Open.*

```
        Cfix8 = 0.756;
        Cfix9 = 0.551;
        Cfix10 = 0.634;

 !DSL ROTATION MATRIX (DERIVE FROM STEP 2)
       NEW (rot11 rot12 rot13 rot14);
       rot11 = 0.6257501;
       rot12 = 0.3709949;
       rot13 = 0.4698927;
       rot14 = -0.5000003;

       NEW (rot21 rot22 rot23 rot24);
       rot21 = 0.7728718;
       rot22 = -0.2262035;
       rot23 = -0.3185918;
       rot24 = 0.5000004;

       NEW (rot31 rot32 rot33 rot34);
       rot31 = -0.08546519;
       rot32 = 0.86112749;
       rot33 = -0.03404132;
       rot34 = 0.49999633;

       NEW (rot41 rot42 rot43 rot44);
       rot41 = -0.06165699;
       rot42 = -0.26392079;
       rot43 = 0.82252130;
       rot44 = 0.50000294;

       !APPLY ROTATION MATRIX TO BETA !since B* = H B H -1 = BHT, so we simplified as BHT here
       !OUTCOME ON GENERAL FACTOR
       NEW (G_OUT);
       G_OUT = b1*rot11 + b2*rot12 + b3*rot13;
       !OUTCOME ON PSYCHOTIC
       NEW (PSY_OUT);
       PSY_OUT = b1*rot21 + b2*rot22 + b3*rot23;
       !OUTCOME ON EXTERNALIZING
       NEW (EXT_OUT);
       EXT_OUT = b1*rot31 + b2*rot32 + b3*rot33;
       !OUTCOME ON INTERNALIZING
       NEW (INT_OUT);
       INT_OUT = b1*rot41 + b2*rot42 + b3*rot43;

OUTPUT:
STAND; TECH1; TECH4; SAMPSTAT; CINTERVAL; MODINDICES;
```

3. Applying a second-order CFA measurement model

To circumvent the issue with second-order CFA model lacking one degree of freedom when estimating associations with exposures/outcomes, we constrained one specific factor to have zero residual and therefore it would have no association with the exposures/outcomes. This method has already been applied in a recently published study[1]. When the higher order loadings were freely estimated, the internalizing factor was near-perfect loaded on the general factor (loading 0.965). So, we constrained the specific internalizing factor to have zero residual and no association with specific internalizing factor was estimated. Same as second-order EFA model, we fixed the factor loadings to the second-order CFA without outcomes. Below is the Mplus script.

Step 1: Derive the factor loadings to the CFA without outcomes

```
TITLE:
        202306_cfa;
DATA:
        FILE = "mgr_mz_use";
VARIABLE:
       NAMES = bpdfm depfm drgfm anxfm alcfm sczfm Sczaffectfm ocdfm ptsdfm ccfm;
```

```
            MISSING=.;
            USEVARIABLES = bpdfm sczfm Sczaffectfm alcfm drgfm ccfm anxfm depfm ocdfm
                           ptsdfm;
            CATEGORICAL = bpdfm sczfm Sczaffectfm alcfm drgfm ccfm anxfm depfm ocdfm ptsdfm;
    ANALYSIS:
            MODEL = nocovariances;
            PROCESS = 4;
            ESTIMATOR = wlsmv;
    MODEL:

            F1 by bpdfm-Sczaffectfm* (s1-s3);
            F2 by alcfm-ccfm* (s4-s6);
            F3 by anxfm-ptsdfm* (s7-s10);
            F1-F3@0 ;

            r1 by F1*(res1);
            r2 by F2*(res2);
            r3 by F3*(res3);
            r1-r3@1;

            g by F1 * (gload1) ;
            g by F2 * (gload2) ;
            g by F3 * (gload3) ;
            g@1 ;

    MODEL CONSTRAINT:
            !FIX F1-F3 VARIANCES AT UNITY
            0 = 1 - (res1^2 + gload1^2) ;
            0 = 1 - (res2^2 + gload2^2) ;
            0 = 1 - (res3^2 + gload3^2) ;
            gload3=1;

    OUTPUT:
    STAND; TECH1; TECH4; SAMPSTAT;CINTERVAL;MODINDICES;
```

Step 2: Derive the beta with factor loadings deriving from step 1

```
    TITLE:
             202306_cfa_esem;
    DATA:
             FILE = "mgr_mz_use";
    VARIABLE:
            NAMES = bpdfm depfm drgfm anxfm alcfm sczfm Sczaffectfm ocdfm ptsdfm ccfm
                    Outcome  BirYearO  eduP lopnrM;
            MISSING=.;
            USEVARIABLES = bpdfm sczfm Sczaffectfm alcfm drgfm ccfm anxfm depfm ocdfm
                           ptsdfm outcome BirYearO  eduP lopnrM;
            CATEGORICAL = bpdfm sczfm Sczaffectfm alcfm drgfm ccfm anxfm depfm ocdfm ptsdfm
                          outcome;
            CLUSTER is lopnrM;
    DEFINE:
            STANDARDIZE BirYearO ;
    ANALYSIS:
           MODEL = nocovariances;
           TYPE = COMPLEX;
            PROCESS = 4;
            ESTIMATOR = wlsmv;
    MODEL:

            F1 by bpdfm-Sczaffectfm* (s1-s3);
            F2 by alcfm-ccfm* (s4-s6);
            F3 by anxfm-ptsdfm* (s7-s10);
            F1-F3@0 ;
```

© 2023 Zhou M et al. *JAMA Network Open.*

```
        r1 by F1*(res1);
        r2 by F2*(res2);
        r3 by F3*(res3);
        r1-r3@1;

        g by F1 * (gload1) ;
        g by F2 * (gload2) ;
        g by F3 * (gload3) ;
        g@1 ;

        !REGRESSIONS
        outcome on r1 (b1) ;
        outcome on r2 (b2) ;
        outcome on g (b3) ;
        outcome on BirYearO (b4) ;
        outcome on eduP (b5) ;

        BirYearO with r1 (r41) ;
        BirYearO with r2 (r42) ;
        BirYearO with g (r43) ;

        eduP with r1 (r51) ;
        eduP with r2 (r52) ;
        eduP with g (r53) ;

MODEL CONSTRAINT:
        !FIX FACTOR LOADINGS FROM STEP 1
          s1 = 0.910;
          s2 = 0.716;
          s3 = 0.819;
          s4 = 0.793;
          s5 = 0.817;
          s6 = 0.578;
          s7 = 0.785;
          s8 = 0.846;
          s9 = 0.584;
          s10 = 0.729;

          res1=0.726;
          res2=0.716;
          res3=0;
          gload1=0.688;
          gload2=0.698;
          gload3=1;

OUTPUT:
STAND; TECH1; TECH4; SAMPSTAT;CINTERVAL;MODINDICES;
```

eReferences 2.

1. Du Rietz E, Pettersson E, Brikell I, et al. Overlap between attention-deficit hyperactivity disorder and neurodevelopmental, externalising and internalising disorders: separating unique from general psychopathology effects. The British Journal of Psychiatry 2021;218(1):35-42. DOI: 10.1192/bjp.2020.152.

© 2023 Zhou M et al. *JAMA Network Open.*
