# Supplementary material for: Intergenerational Transmission of Psychiatric Conditions and Psychiatric, Behavioral, and Psychosocial Outcomes in Offspring
Source: JAMA Netw Open. 2023 Dec 20;6(12):e2348439. doi: 10.1001/jamanetworkopen.2023.48439 (PMC10733806; doi:10.1001/jamanetworkopen.2023.48439)
Supplement: Supplement 2. — Data Sharing Statement [file jamanetwopen-e2348439-s002.pdf]

Data Sharing Statement

Zhou. Intergenerational Transmission of Psychiatric Conditions and Psychiatric, Behavioral, and Psychosocial Outcomes in Offspring. *JAMA Netw Open*. Published December 20, 2023. doi:10.1001/jamanetworkopen.2023.48439

Data
Data available: Yes
Data types: Deidentified participant data
How to access data: mengping.zhou@ki.se
When available: With publication

Supporting Documents
Document types: Statistical/analytic code
How to access documents: mengping.zhou@ki.se
When available: With publication

Additional Information
Who can access the data: researchers whose proposed use of the data has been approved
Types of analyses: for any purpose or for a specified purpose
Mechanisms of data availability: with a signed data access agreement
